# Population Structure and Transmission Dynamics of *Plasmodium vivax* in the Republic of Korea Based on Microsatellite DNA Analysis

**DOI:** 10.1371/journal.pntd.0001592

**Published:** 2012-04-03

**Authors:** Moritoshi Iwagami, Megumi Fukumoto, Seung-Young Hwang, So-Hee Kim, Weon-Gyu Kho, Shigeyuki Kano

**Affiliations:** 1 Department of Tropical Medicine and Malaria, Research Institute, National Center for Global Health and Medicine, Shinjuku, Tokyo, Japan; 2 Graduate School of Comprehensive Human Sciences, University of Tsukuba, Tsukuba, Ibaraki, Japan; 3 Department of Parasitology, College of Medicine, Inje University, Busanjin-gu, Busan, Korea; 4 Department of Malariology, College of Medicine, Paik Institute of Clinical Research, Inje University, Busanjin-gu, Busan, Korea; Ege University, Turkey

## Abstract

**Background:**

In order to control malaria, it is important to understand the genetic structure of the parasites in each endemic area. *Plasmodium vivax* is widely distributed in the tropical to temperate regions of Asia and South America, but effective strategies for its elimination have yet to be designed. In South Korea, for example, indigenous vivax malaria was eliminated by the late 1970s, but re-emerged from 1993. We estimated the population structure and temporal dynamics of transmission of *P. vivax* in South Korea using microsatellite DNA markers.

**Methodology/Principal Findings:**

We analyzed 255 South Korean *P. vivax* isolates collected from 1994 to 2008, based on 10 highly polymorphic microsatellite DNA loci of the *P. vivax* genome. Allelic data were obtained for the 87 isolates and their microsatellite haplotypes were determined based on a combination of allelic data of the loci. In total, 40 haplotypes were observed. There were two predominant haplotypes: H16 and H25. H16 was observed in 9 isolates (10%) from 1996 to 2005, and H25 in 27 (31%) from 1995 to 2003. These results suggested that the recombination rate of *P. vivax* in South Korea, a temperate country, was lower than in tropical areas where identical haplotypes were rarely seen in the following year. Next, we estimated the relationships among the 40 haplotypes by eBURST analysis. Two major groups were found: one composed of 36 isolates (41%) including H25; the other of 20 isolates (23%) including H16. Despite the low recombination rate, other new haplotypes that are genetically distinct from the 2 groups have also been observed since 1997 (H27).

**Conclusions/Significance:**

These results suggested a continual introduction of *P. vivax* from other population sources, probably North Korea. Molecular epidemiology using microsatellite DNA of the *P. vivax* population is effective for assessing the population structure and transmission dynamics of the parasites - information that can assist in the elimination of vivax malaria in endemic areas.

## Author Summary

Vivax malaria is widely prevalent, mainly in Asia and South America with 390 million reported cases in 2009. Worldwide, in the same year, 2.85 billion people were at risk. *Plasmodium vivax* is prevalent not only in tropical and subtropical areas but also in temperate areas where there are no mosquitoes in cold seasons. While most malaria researchers are focusing their studies on the parasite in tropical areas, we examined the characteristics of *P. vivax* in South Korea (temperate area) temporally, using 10 highly polymorphic microsatellite DNA (a short tandem repeat DNA sequence) in the parasite genome, and highlighted the differences between the tropical and temperate populations. We found that the South Korean *P. vivax* population had low genetic diversity and low recombination rates in comparison to tropical *P. vivax* populations that had been reported. We also found that some of the parasite clones in the population were changing from 1994 to 2008, evidence suggesting the continual introduction of the parasite from other populations, probably from North Korea. Polymorphic DNA markers of the *P. vivax* parasite are useful tools for estimating the situation of its transmission in endemic areas.

## Introduction

*Plasmodium vivax, the second most prevalent species of the human malaria parasite, is widely distributed around the world, especially in Asia and South America; it ranges from tropical to temperate areas [1], [2]. In these countries, the proportion of P. falciparum cases is gradually decreasing due to the impact of global malaria control programs such as “The Roll Back Malaria Partnership” and “The Global Fund to Fight AIDS, Tuberculosis and Malaria” as well as local control programs. In contrast, the proportion of P. vivax cases is gradually increasing [1], and therefore deserves more attention than it has previously received [3].*

Understanding the genetic characteristics of the malaria parasite population is important for monitoring the transmission pattern and evaluating the effectiveness of malaria control in endemic areas [4]–[7]. Recently, the population structure and transmission dynamics of *P. vivax* have been reported in some tropical and subtropical areas where the parasites are prevalent throughout the year or seasonally prevalent but not discontinuous during the year [8]–[13]. However, little is known about these characteristics in temperate areas where vivax malaria is only seasonally prevalent and discontinuous during the year.

**Figure 1:**
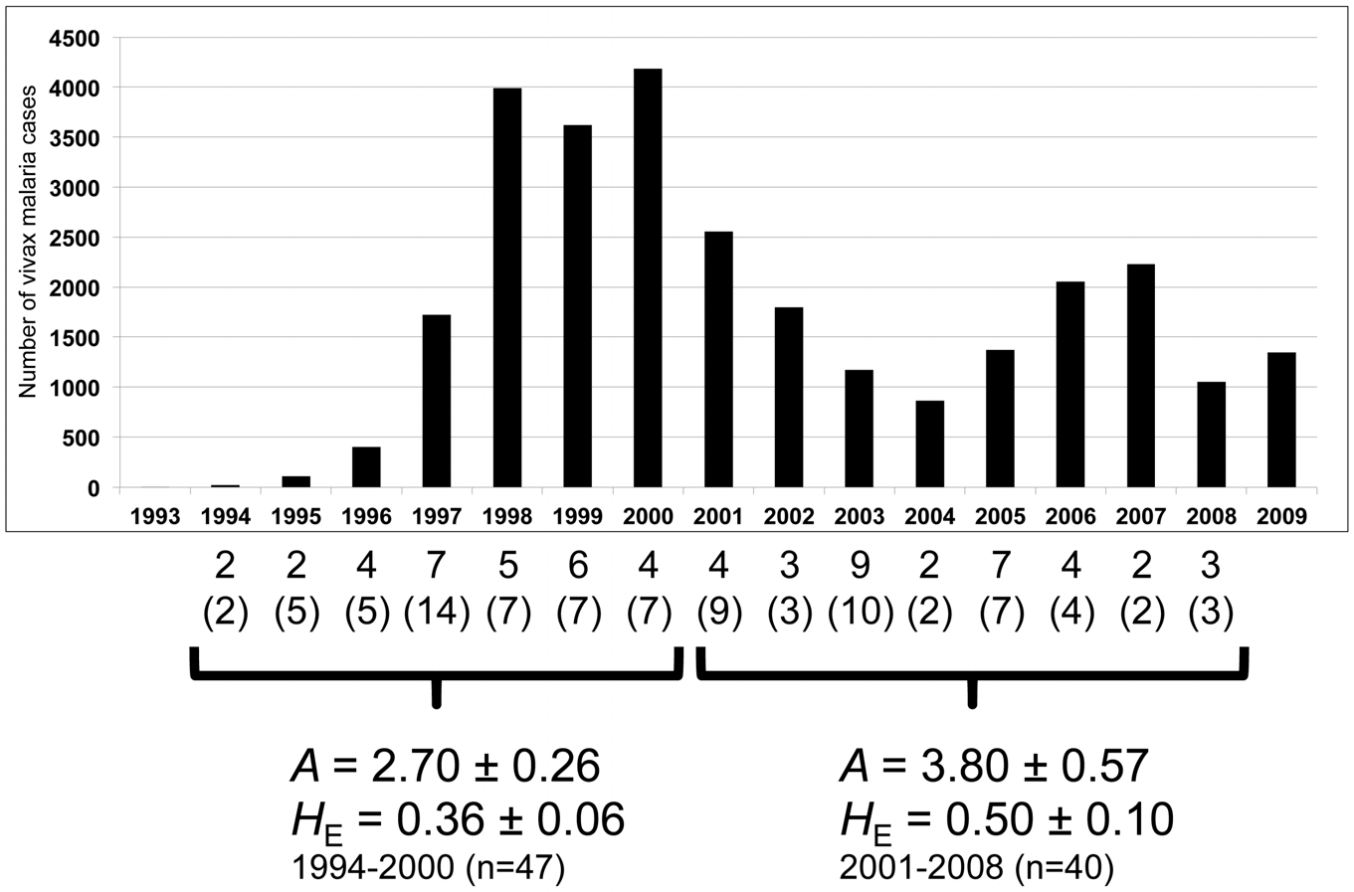
Genetic diversity of the *P. vivax* population in South Korea. *A*: Average number of the alleles ± SE, *H_E_*: Average expected heterozygosity ± SE. Numbers and numbers in parentheses represent number of haplotypes and isolates observed each year, respectively. The graph was made based on numbers of reported vivax malaria cases in South Korea. n: represents the number of isolates. The data were obtained from the *World Malaria Report 2010* (WHO) [1].

In the Republic of Korea (South Korea), which is in the temperate zone of the continent of Asia, indigenous vivax malaria had been successfully eliminated by the late 1970s thanks to an effective program conducted by the National Malaria Eradication Service of the South Korean government with the support of the WHO [14]–[16], but has re-emerged since 1993 [17]. At the beginning of the re-emergence, the patients were only South Korean soldiers, veterans, and soldiers from the US military who were serving in the border area between North and South Korea in the western Demilitarized Zone (DMZ) [18]–[20]. Gradually, however, the number of infected civilians who lived in or near the area increased [18], suggesting local transmission of *P. vivax* between humans and *Anopheles* mosquitoes in the country. The number of vivax malaria cases increased steadily until 2000 (4,183 cases), then began to decrease gradually until 2004 (864 cases) (Fig. 1) [1], [16]. In spite of continuous malaria control measures implemented by the South Korean government, the numbers of reported cases fluctuated between 1,000 and 2,000 cases per year from 2005 to 2009 [1]. The WHO reports that vivax malaria was more prevalent in the Democratic People's Republic of Korea (North Korea), where there were 296,540 cases in 2001 and 14,845 cases in 2009 [1], [19].

We previously conducted genetic epidemiological surveys of the *P. vivax* population in South Korea using DNA sequences of some antigenic molecules of the parasite (circumsporozoite protein, Duffy binding protein, apical membrane antigen 1, merozoite surface protein-1) and found that there were 2 genotypes in the country's parasite population [21]–[25]. The advantage of using antigenic molecules of the parasites for genetic epidemiology is that they could be vaccine candidates; however such antigenic molecules are under strong selective pressure from the host immune system, so the variation in the molecules might be biased due to this [26]. In previous studies, the isolates that were used were collected from vivax malaria patients in a single year so temporal changes in the parasite population could not be examined. In the present study, we examined the population structure and the transmission dynamics of *P. vivax* in South Korea temporally using 10 highly polymorphic neutral DNA markers of the parasite collected from 1994 to 2008 and compared these characteristics with those reported in tropical and subtropical areas. Based on these data, we provide a possible explanation as to why it has not been possible to eliminate vivax malaria in South Korea in spite of a continuous governmental effort.

## Methods

### Materials

A total of 255 *P. vivax* samples isolated from South Korean soldiers or veterans who had served in the DMZ from 1994 to 2008 were used in this study. These patients were also diagnosed by microscopic examination of peripheral blood smears when they contracted malaria. The patient blood samples were collected and preserved at −30°C until use. This study was performed according to the Ethical Guidelines for Clinical Research issued by the Ministry of Health, Labour and Welfare of Japan on July 31, 2008, and the Ethical Guidelines for Epidemiological Research issued by the Ministries of Health, Labour and Welfare, and of Education, Science, Culture, and Sports of Japan on December 1, 2008. Because of the long-term prior collection of widely distributed samples, written or oral informed consent from the patients for the specific purpose of this study could not be obtained at each sample collection. However, no author of the study was involved in gathering patient samples and the individual information of the donors was disconnected from the authors. Thus, all the samples were anonymized, and indeed it is most unlikely that the results obtained from the analysis of the isolated parasites would result in a breach of donor privacy.

### DNA extraction

Parasite DNA was extracted from frozen whole blood samples by phenol-chloroform extraction after proteinase K digestion [27] or by QIAamp DNA Mini Kit (Qiagen, Valencia, CA, USA).

### Genotyping by polymerase chain reaction (PCR)

Ten microsatellite DNA loci were amplified by PCR. The loci were as follows: MS1 (chromosome 3), MS4 (chromosome 6), MS5 (chromosome 6), MS6 (chromosome 11), MS7 (chromosome 12), MS8 (chromosome 12), MS9 (chromosome 8), MS12 (chromosome 5), MS15 (chromosome 5) and MS20 (chromosome 10). The PCR primer sets and amplification conditions were consistent with the protocol of Karunaweera et al. [28]. Sizes of fluorescently-labeled PCR products were measured on an Applied Biosystems Prism Genetic Analyzer 3130*xl* using GeneMapper^(R)^ version 4.1 with a 500 ROX size standard (Applied Biosystems, CA, USA).

Amplified different-sized PCR products using the same primer sets were considered to be individual alleles within a locus, as size variation among isolates is consistent with the repeat number in a microsatellite locus [5]. The electropherogram shows peak profiles for the microsatellite loci, based on the fluorescence intensity of the labeled PCR products in this analysis. Multiple alleles per locus were scored if minor peaks were taller than at least one-third the height of the predominant allele for each locus. Multiple-genotype infections (MGIs) were defined as those in which at least one of the 10 loci contained more than one allele [5].

### Population genetic analyses

Population genetic analyses were performed based on allele frequencies of the 10 microsatellite loci of the population. The level of genetic diversity of the *P. vivax* population in South Korea was assessed by allele number per locus (*A*) and expected heterozygosity (*H*_E_). *H*_E_ values for each locus were calculated using *H*_E_ = [n/(n−1)] [1−Σ*p*_i_^2^], where n corresponds to the number of isolates examined and *p*_i_ is the frequency of the i^th^ allele. The statistical differences among those values were evaluated by Welch's t-test.

Multilocus linkage disequilibrium (LD) was assessed using the standardized index of association (*I*_A_^S^) [29], [30]. This analysis was performed using the LIAN 3.5 Web interface [31]. *I*_A_^S^ was calculated using the formula *I*_A_^S^ = (*V*_D_/*V*e−1)/(*l*−1) with permutation testing of the null hypothesis of complete linkage equilibrium (*I*_A_^S^ = 0), where *V*_D_ is the observed mismatch variance, *V*e is the expected mismatch variance, and *l* is the number of examined loci. Significances of the observed *I*_A_^S^ values were calculated by Monte-Carlo simulation, using 10,000 random permutations of the data. This statistic is a variation of the method proposed by Maynard-Smith et al [29]. The results were standardized by the number of loci, to enable a comparison of different data sets [30]. This test was applied to the data sets from each population in two ways. First, the mixed-clone infections were excluded so that only the single-clone infections were analyzed, giving absolute confidence in the haplotype profile. Second, any multilocus genotype found in more than one isolate was only counted once in the analysis, i.e. unique haplotypes only, reducing the sample size slightly and thereby removing the possible effect of recent epidemic expansion of particular clones [5].

Microsatellite haplotypes of the isolates were determined based on a combination of the allelic data of the 10 loci. The relationships among the haplotypes were estimated by eBURST analysis [32].

## Results

The allelic data of the 10 microsatellite loci were obtained from 87 of the 255 (34%) isolates that were used in the study. They were not available in the remaining 168 isolates (66%) due to failure in acquiring PCR products of some loci by PCR-based genotyping. Failure was possibly due to there being only a small amount of DNA for PCR amplification or to the DNA being of low quality after multiple times of freeze-thaw.

**Figure 2:**
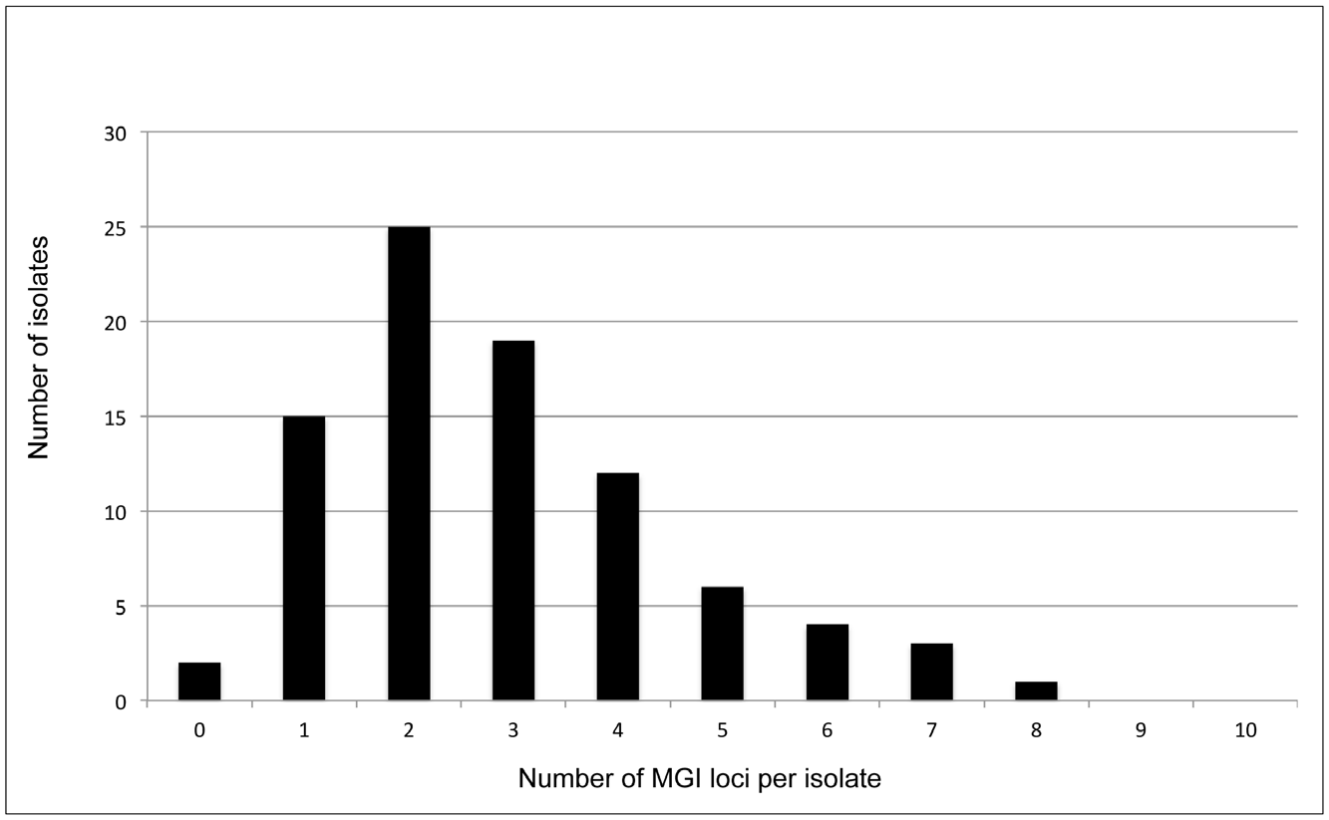
Frequency of MGI loci per isolate. Zero in the x-axis indicates that no MGI loci were observed in a particular isolate, that is, it represents a single clone infection isolate.

When different sizes of alleles were observed in one locus, we regarded this as multiple genotype infections (MGIs). MGIs were observed in some of the 10 microsatellite loci in 85 of the 87 isolates (97.7%). The frequencies of MGIs varied among the 10 loci (0.00 to 0.84; average: 0.29) (Table 1). We also examined the number of MGI loci per isolate. In the 87 isolates with 10 loci, the highest frequency of MGI loci per isolate was 2 (25 isolates) and the frequencies decreased gradually according to the increase in the number of MGI loci (Fig. 2). The highest number of MGI loci per isolate was 8 (one isolate). The major alleles in each locus were used for population genetic analysis.

**Table 1:** Multiple Genotype Infection rate per locus of the *P. vivax* population in South Korea.

Locus	No. of MGI isolates	MGI rate (%)
MS1	9	10.3
MS4	52	59.8
MS5	6	6.9
MS6	3	3.4
MS7	73	83.9
MS8	35	40.2
MS9	10	11.5
MS12	38	43.7
MS15	0	0.0
MS20	27	31.0
Average	25.3	29.1

MGI: Multiple genotype infection. Sample size: 87 isolates.

### Genetic diversity

In the 10 loci, the number of alleles (*A*) for each locus was 2 to 7 (average: 4.3). The expected heterozygosity (*H*_E_) for each of these loci was 0.05 to 0.66 (average: 0.43) (Table 2).

**Table 2:** Genetic diversity of *P. vivax* populations in South Korea, Sri Lanka and the Brazilian Amazon.

Locus	Chr	Core repeat sequence in the	Type of region	South Korea (n = 87)		Sri Lanka (n = 25)		Brazil (n = 99)	
	(ID)	Salvador-I strain		*A*	*H*_E_	A	*H*_E_	A	*H*_E_
MS1	3 (CM000444)	(GAA)_11_	Repeat region	5	0.61	7	0.77	5	0.69
MS4	6 (CM000447)	(AGT)_18_	Gene coding a hypothetical protein	3	0.55	6	0.75	6	0.69
MS5	6 (CM000447)	CCTCTT(CCT)_11_	Gene coding a hypothetical protein	6	0.62	6	0.81	16	0.87
MS6	11 (CM000452)	(TCC)_2_(TCT)_3_(CCT)_2_(TCC)_2_ GCTTCT(TCC)_10_	Repeat region	7	0.66	6	0.83	8	0.66
MS7	12 (CM000453)	(GAA)_9_	Between two genes*	6	0.58	6	0.65	3	0.48
MS8	12 (CM000453)	(CAG)_2_(CAA)_11_	Gene coding a 3′-5′ exonuclease domein containing protein	3	0.05	11	0.84	16	0.84
MS9	8 (CM000449)	(GGA)_18_	Gene coding a hypothetical protein	3	0.57	6	0.80	7	0.78
MS12	5 (CM000446)	(TTC)_10_(TGC)_4_	Repeat region	3	0.07	6	0.81	8	0.74
MS15	5 (CM000446)	(TCT)_10_	Gene coding a CW-type zinc finger domain containing protein	5	0.54	8	0.86	16	0.74
MS20	10 (CM000451)	(GAA)_11_GAG(GAA)_13_(CAA)_4_GAA(CAA)_5_	Gene coding a tryptophan-rich antigen	2	0.12	13	0.91	11	0.82
Average				4.3	0.43	7.5	0.80	9.6	0.73

Chr: Chromosome No, ID represents Chromosome ID of *P. vivax* Salvador 1 in the GenBank Database, A: Number of alleles, *H*E: Expected heterozygosity, Data of Sri Lankan and Brazilian populations were obtained from Karunaweera, et al. (2007) [28] and Orjuela-Sánchez, et al. (2009) [33], respectively. n: Number of isolates.

*: Two genes are encoding a hypothetical protein and a merozite surface protein-7.

Next, the *P. vivax* population was divided into 2 groups: one comprised of the 47 isolates collected from 1994 to 2000 when the numbers of vivax malaria cases increased; the other comprised of the 40 isolates collected from 2001 to 2008, when the numbers of cases decreased until 2004 and then increased slightly. The level of genetic diversity was reassessed for each group. For the first group, the averages ± SE of *A* and *H*_E_ were 2.70±0.26 and 0.36±0.06, respectively. For the second group, the averages ± SE of *A* and *H*_E_ were 3.80±0.57 and 0.50±0.10, respectively (Fig. 1). The levels of genetic diversity were relatively higher in the second group, with *P* values at 0.11 and 0.24 for average *A* and average *H*_E_, respectively.

**Figure 3:**
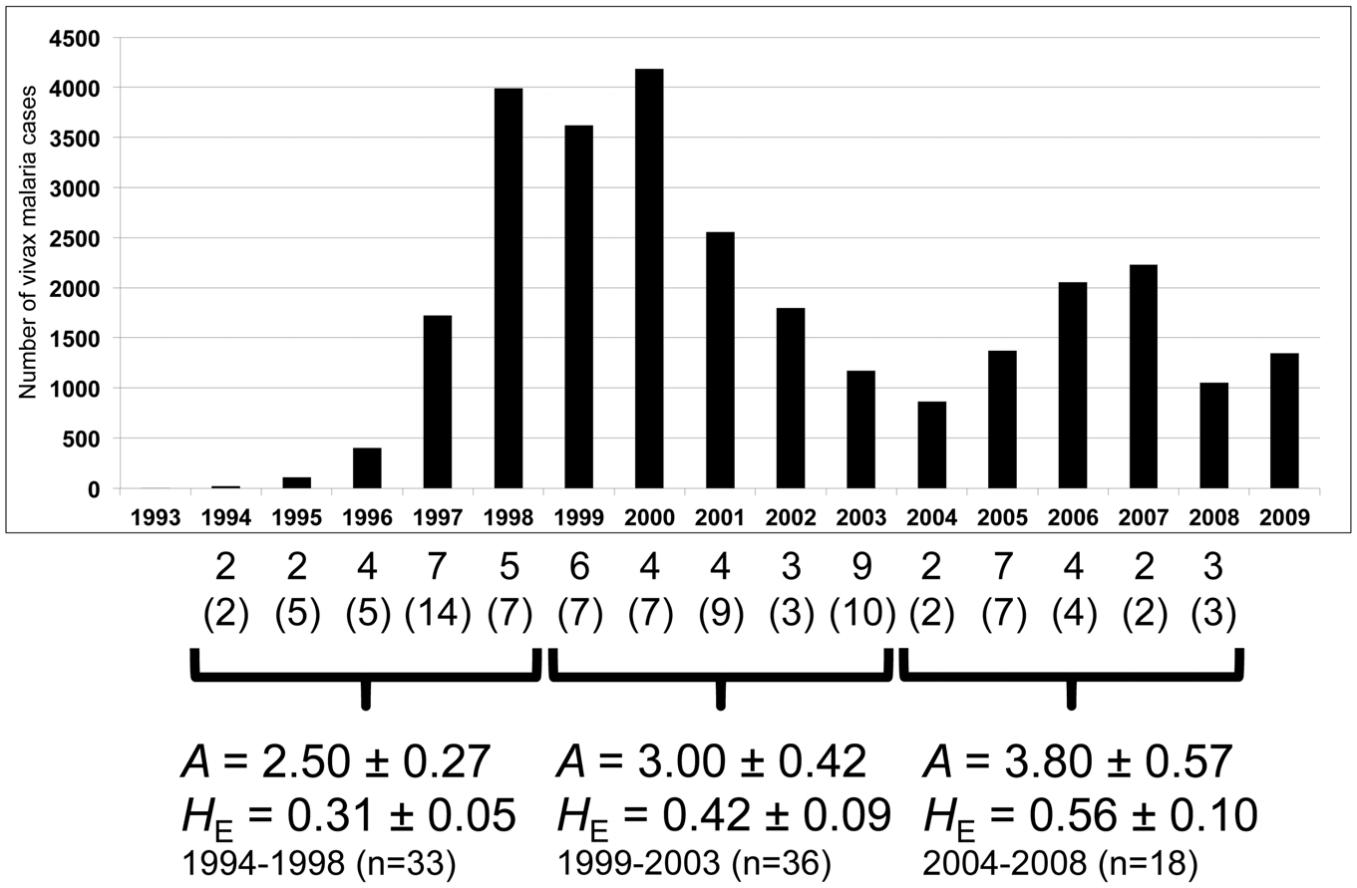
Genetic diversity of the *P. vivax* population in South Korea. *A*: Average number of the alleles ± SE, *H_E_*: Average expected heterozygosity ± SE. Numbers and numbers in parentheses represent the number of haplotypes and isolates observed each year, respectively. The graph was made based on numbers of reported vivax malaria cases in South Korea. n: represents the number of isolates. The data were obtained from the *World Malaria Report 2010* (WHO) [1].

Furthermore, we also divided the population into 3 groups, each covering 5-year periods: 1994 to 1998 (33 isolates), 1999 to 2003 (36 isolates) and 2004 to 2008 (18 isolates). The level of genetic diversity was reassessed for each group (Fig. 3). For the first group, the averages ± SE of *A* and *H*_E_ were 2.50±0.27 and 0.31±0.05, respectively. For the second group, the averages ± SE of *A* and *H*_E_ were 3.00±0.42 and 0.42±0.09, respectively. For the third group, the averages ± SE of *A* and *H*_E_ were 3.80±0.57 and 0.56±0.10, respectively. The levels of genetic diversity gradually increased with *P* values at 0.06 and 0.05 if we compared the difference of the average *A* between the first group (1994–1998) and the third group (2003–2008) and the difference of the average *H*_E_ between the first and the third group, respectively.

### Multilocus linkage disequilibrium (LD)

Likewise, the analysis of genetic diversity, *I*_A_^S^ values were also calculated for the two populations: one comprised the isolates collected from 1994 to 2000 and the other comprised the isolates collected from 2001 to 2008, with permutation testing of the null hypothesis of *I*_A_^S^ = 0 (equilibrium of multilocus frequencies) (Table 3). When the single-clone haplotype was used in the analysis, the *I*_A_^S^ values of the former (1994–2000) and the latter (2001–2008) were 0.529 and 0.218, respectively, whereas when the unique haplotypes were used in the analysis, those of the former and the latter were 0.346 and 0.173, respectively. Significant linkage disequilibrium was observed in both populations (*P*<0.001).

**Table 3:** Multilocus linkage disequilibrium in the two *P. vivax* populations.

Population	Single Clones		Unique Haplotypes Only	
	No.	*I_A_^S^*	No.	*I_A_^S^*
1994–2000	47	0.529***	19	0.346***
2001–2008	40	0.218***	27	0.173***

Single clones show all haplotypes in single-clone infections. Unique haplotypes show haplotypes excluding duplicates of any multiply represented infection. No. indicates the number of isolates for each measure.

***: *P*<0.001.

Similar to the analyses of genetic diversity, we also divided the population into 3 groups covering 5-year periods: 1994 to 1998 (33 isolates), 1999 to 2003 (36 isolates) and 2004 to 2008 (18 isolates). The *I*_A_^S^ values were also calculated for each group. When the single-clone haplotype was used in the analysis, the *I*_A_^S^ values of the first (1994–1998), the second (1999–2003) and the third (2004–2008) groups were 0.584, 0.315 and 0.140, respectively, whereas when the unique haplotypes were used in the analysis, those of the first, the second and the third were 0.408, 0.231 and 0.153, respectively (Table 4). Significant linkage disequilibrium was observed in both populations (*P*<0.001).

**Table 4:** Multilocus linkage disequilibrium in the two *P. vivax* populations.

Population	Single Clones		Unique Haplotypes Only	
	No.	*I_A_^S^*	No.	*I_A_^S^*
1994–1998	33	0.584***	13	0.408***
1999–2003	36	0.315***	17	0.231***
2004–2008	18	0.140***	18	0.153***

Single clones show all haplotypes in single-clone infections. Unique haplotypes show haplotypes excluding duplicates of any multiply represented infection. No. indicates the number of isolates for each measure.

***: *P*<0.001.

### Haplotypes and the relationships among the haplotypes

Microsatellite haplotypes of the 87 isolates were determined based on a combination of the allelic data of the 10 microsatellite loci; 40 haplotypes (H1–H40) were observed (Table 5). There were 2 major haplotypes (H16 and H25): H16 was observed in 9 isolates (10%) out of the 87 isolates in samples collected from 1996 to 2005; H25 was observed in 27 isolates (31%) out of the 87 isolates in samples collected from 1995 to 2003. H16 and H25 share only 3 alleles in the loci, MS8, MS12 and MS20, but those in 7 other loci were different from each other.

**Table 5:** Forty haplotypes of the *P. vivax* population in South Korea based on 10 microsatellite DNA loci.

H	Year															Total
	1994	1995	1996	1997	1998	1999	2000	2001	2002	2003	2004	2005	2006	2007	2008	
H1												*1*				*1*
H2	*1*															*1*
H3							*1*									*1*
H4			*1*													*1*
H5												*1*				*1*
H6													*1*			*1*
H7															*1*	*1*
H8											*1*					*1*
H9										*1*		*1*				*2*
H10										*1*			*1*			*2*
H11														*1*		*1*
H12										*1*						*1*
H13				*1*												*1*
H14				*1*				*1*								*2*
H15							*1*									*1*
**H16**			**1**		**1**	**2**	**2**	**2**				**1**				**9**
H17				*1*												*1*
H18						*1*			*1*							*2*
H19															*1*	*1*
H20	*1*										*1*					*2*
H21			*1*													*1*
H22					*1*											*1*
H23										*1*						*1*
H24		*1*		*1*	*1*											*3*
**H25**		**4**	**2**	**8**	**3**	**1**	**3**	**4**		**2**						**27**
H26				*1*								*1*				*2*
H27				*1*												*1*
H28						*1*										*1*
H29										*1*						*1*
H30										*1*						*1*
H31														*1*		*1*
H32												*1*				*1*
H33					*1*			*2*	*1*							*4*
H34						*1*										*1*
H35						*1*										*1*
H36													*1*			*1*
H37										*1*						*1*
H38									*1*	*1*			*1*			*3*
H39												*1*				*1*
H40															*1*	*1*
Total	2	5	5	14	7	7	7	9	3	10	2	7	4	2	3	87

H: Haplotype, Numbers show number of isolates. Total: Total No. of isolates, Two predominant haplotypes, and H25 are highlighted in bold and the other haplotypes are highlighted in italic.

**Figure 4:**
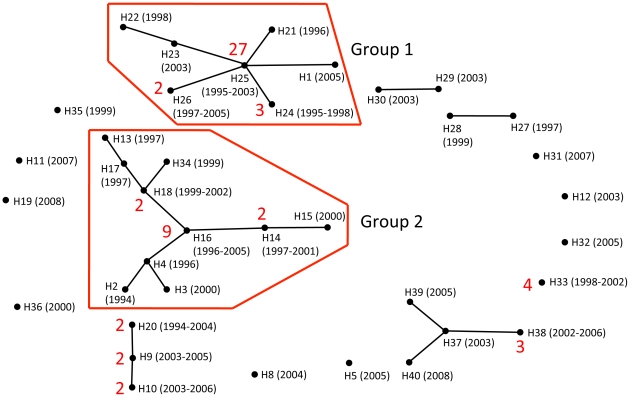
Relationships among the 40 haplotypes of *P. vivax* (n = 87) in South Korea estimated by eBURST analysis. Relationships among 40 microsatellite haplotypes in the 87 isolates collected in South Korea as defined by eBURST analysis [32]. H1, H2, … H40 represent the microsatellite haplotype. Red numbers represent the number of isolates that showed the haplotype. When the haplotype was found in only one isolate, the red number was omitted. Numbers in parentheses represent the year the haplotype was observed. H6 and H7 were included in the analysis but were classified into a different group because they were considerably different from others and were omitted from Figure 4.

The relationships among the 40 haplotypes were estimated by eBURST analysis [32] with the following criterion: when 2 isolates shared more than 7 identical loci out of the 10 loci, they were connected with a branch (Fig. 4). Again, two major groups were found: Group 1 was composed of 36 isolates (41%) including the isolates with H25; Group 2 was composed of 20 isolates (23%) including those with H16. Some new or isolated haplotypes, namely H5, H6, H7, H8, H11, H12, H19, H31, H32, H33, H35, H36, that were not included in the 2 major groups or connected to any other haplotypes, have also been observed since 1998. H6 and H7 were not shown in Figure 2 because these haplotypes were quite different from the other haplotypes.

## Discussion

This is the first 15-year-long longitudinal study on *P. vivax* population genetics using highly polymorphic neutral markers. The present study demonstrated that the level of genetic diversity of the *P. vivax* population in South Korea was remarkably lower than the levels in tropical and subtropical areas reported by Karunaweera et al. [28] and Orjuela-Sánchez et al. [33] (Table 2). The 10 microsatellite loci used in the present study were a subset of the 14 loci used in the previous studies by other groups [28], [33]. Imwong et al. also reported that the mean values of *H*_E_ of *P. vivax* populations from Thailand (n = 28), India (n = 27) and Colombia (n = 27) were 0.77, 0.76 and 0.64, respectively [11], using 11 other microsatellite loci in the genome. These values reported by Imwong et al. were also higher than those in South Korea.

Sample size (n) and sampling conditions such as the size of sampling area and the length of sampling period may affect levels of genetic diversity of living organisms. In comparison to other studies the sample size of the present study (n = 87) was relatively large and the sampling period (15 years: from 1994 to 2008) was relatively long [11], [28], [33]. Generally, one would expect to see an increase in the level of genetic diversity when these conditions (a large number of samples and a long sampling period) are present. However, the South Korean *P. vivax* population showed low levels of genetic diversity, suggesting that the effective size of the re-emerged *P. vivax* population in South Korea might be small.

Microsatellite variation is strongly dependent on the length of repeat arrays [34]. Studies of numerous organisms have shown higher levels of variation in loci with long repeat arrays than those with short repeat arrays [35]. In the present study, however, even the locus with a long repeat array (MS20) showed low levels of variation in the South Korean population. Also in the same population, MS8 and MS12 showed low levels of variation, although the loci were not short repeat arrays. The genetic diversities of the loci from Sri Lankan and Brazilian populations (Table 2) were higher than those from the South Korean population. Therefore, the uniqueness of the diversity would not be solely dependent on the characteristics of the loci. In fact, mutations of microsatellite loci are generally considered to be neutral. However, if the loci are in a certain gene or close to a certain gene, the mutation may not be strictly neutral. Indeed, 6 of the 10 loci examined in this study (MS4, MS5, MS8, MS9, MS15, MS20) were in a gene coding a hypothetical protein or a known protein (Table 2). One of the 10 loci (MS7) was between a gene coding a hypothetical protein and a gene coding a merozoite surface protein-7 which is expected to be under strong selective pressure. Therefore, the mutation of those 7 loci may not be strictly neutral. The allelic data suggested that the frequencies of strand-slippage events of the microsatellite loci during mitotic replication in the South Korean *P. vivax* population were very low because identical alleles in the known loci have been found for 10 years or longer in this population.

Multiple genotype infection (MGI) is one of the important indexes of population genetics and epidemiology of malaria parasites because MGI is the first step in recombination of the parasite genome between different clones. In the case of *P. falciparum*, the rate of MGIs per population is basically associated with the endemicity [4], [5]. That is, the MGI rate of *P. falciparum* population is higher in high transmission areas and lower in low transmission areas. However, this is not the case with *P. vivax* populations because high MGI rates were observed among the *P. vivax* populations in low transmission areas [11], [12]. This feature could be attributed to relapse owing to hypnozoites in the liver of a vivax malaria patient. Although MGI is an important index, the methods or criteria of determining MGI is problematic. When any locus of the 10 loci showed more than 1 allele, we regarded the isolate as an example of MGI. Using this method, 85 (97.7%) out of the 87 isolates were MGIs. Focusing on each locus, the MGI rate per locus varied from 0.0% to 83.9% (average 29.1%) (Table 1). Focusing on the number of MGI loci per isolate, we found an interesting distribution pattern, similar to an F-distribution (Fig. 2). In the present study, the highest frequency of MGI loci per isolate was 2 (found in 25 isolates). The frequency decreased gradually, that is, 3 MGI loci; 19 isolates, 4 MGI loci; 12 isolates, 5 MGI loci; 6 isolates, and so on. We suspect that this distribution pattern may vary in each endemic area with different endemicity.

In the case of *P. falciparum* populations, the levels of genetic diversity are normally associated with the levels of malaria endemicity. That is, the levels of genetic diversity of the parasite populations are higher in high transmission areas and lower in low transmission areas [4], [5], although some exceptions have been reported [36].

We suspect that there will also be some association between the levels of genetic diversity and the levels of malaria endemicity in *P. vivax* populations, even though the correlation between these factors is not clearly understood at the time of writing. In *P. vivax* populations, the levels of genetic diversity tend to be high even in low transmission areas [11]–[13], [28]. This tendency can likely be attributed to unique biological features of *P. vivax*, such as early gametocytogenesis and relapse. Early gametocytogenesis may enhance the efficiency of transmission to *Anopheles* mosquitoes, allowing transmission to occur before symptoms appear – or, more importantly, before antimalarial drugs are administered. Relapses may also enhance the transmission and increase the genetic diversity of *P. vivax* populations, because the relapse will increase the probability of the coexistence of multiple genotype clones in a single patient, which are subsequently sucked up by an *Anopheles* mosquito in a single meal. Thus, the levels of genetic diversity of *P. vivax* populations could be higher than those observed in *P. falciparum* populations even in low transmission areas.

In the present study, the levels of genetic diversity of the South Korean population between 1994 and 2000 (when the number of malaria cases increased) were relatively lower than the levels of genetic diversity between 2001 and 2008 (when the number decreased). On the contrary, the levels of multilocus LD in the population between 1994 and 2000 were relatively higher than those between 2001 and 2008. These results suggested that the latter population was more genetically diverse and had less inbreeding. Furthermore, we divided the population into 3 groups each covering 5-year periods (1994–1998, 1999–2003 and 2004–2008) and reexamined the levels of genetic diversity and multilocus LD. Then, we again observed that the levels of genetic diversity in the populations had gradually increased, whereas the levels of multilocus LD had gradually decreased even though there was still strong multilocus LD in the most recent population (2004–2008). This result was surprising to us because we expected that the effective population size of the latter population would have decreased due to the reduction in the number of alleles in the population. However, this was not the case. In the South Korean populations, the association between the diversity and the endemicity of the *P. vivax* population is elusive.

There are, however, at least two possible explanations for this result. One is that the levels of genetic diversity of the *P. vivax* population increased in North Korea from 2001 to 2002, while the number of vivax malaria cases was very high (296,540 cases in 2001, 241,190 cases in 2002) [1]. Some of the isolates might have then been introduced to South Korea from North Korea by *Anopheles* mosquitoes. The other possible explanation is that the genetic diversity began accumulating in the South Korean population after the re-emergence in 1993. If the latter hypothesis is correct then the malaria control program conducted by the South Korean government might not have affected the parasite population structure.

One of the clear differences between the *P. vivax* population in South Korea and populations in tropical and subtropical areas is the pattern of transmission: in South Korea, vivax malaria is seasonally prevalent with a peak during July and August and no transmission in the winter season [37] and very long incubation periods with 8 to 13 months [14], suggesting that the chance for the recombination of the genome is limited to specific time periods within the year, possibly once or at most twice a year. In fact, we found strong LD in the South Korean population, suggesting that the frequency of recombination in this population would be very limited. However, these results might be associated with the location of the examined MS DNA loci: the 6 loci are in a gene coding a protein and another locus is between 2 genes coding respective proteins. In tropical and subtropical areas, on the other hand, vivax malaria is prevalent throughout the year, and thus recombination may occur throughout the year; this would lead to an increase in the levels of genetic diversity in tropical and subtropical areas. Indeed, in the populations from the Brazilian Amazon, identical haplotypes were rarely observed two years in a row, even in the same endemic area [12]. This would suggest that frequent recombinations occurred between the clones in the population.

The present study showed evidence of a low recombination rate and low frequencies of strand-slippage events of the microsatellite loci during mitotic replication in the *P. vivax* population of South Korea in comparison to populations in tropical and subtropical areas [12], and demonstrated that the 2 dominant haplotypes (H16 and H25) had been transmitting for several years (H16; 1996, 1998–2001, 2005, H25; 1995–2001, 2003) (Table 5). This continuous existence of the same haplotypes for several years is definitive evidence of a low recombination rate in the South Korean *P. vivax* population.

This continuous existence of the same haplotypes could be explained by a local adaptation to vector species. According to Joy et al. [38], for example, *P. vivax* in southern Mexico was genetically differentiated into 3 populations. They suggested that this differentiation would be the result of adaptation to different *Anopheles* species. On the other hand, in South Korea, *Anopheles sinensis* is a main vector of *P. vivax* and the other *Anopheles* species are very minor. Therefore, continuous existence of the predominant haplotypes could not be explained by a local adaptation to certain vector species in this country. There might be some other advantages of these haplotypes, or simply, the variation in the *P. vivax* population on the Korean peninsula had been very small owing to an effective national eradication program conducted by the National Malaria Eradication Service under the operation of the South Korean government with the support of the WHO in the 1970s [14]–[16].

Although the predominant haplotypes (H16 and H25) and their relatives had been transmitting in the DMZ for a long time, their transmission ended in 2005. We speculate that these predominant haplotypes were probably eliminated by the malaria control programs conducted by the North Korean government. In fact, according to the WHO World Malaria Report 2010, the number of vivax malaria cases in North Korea decreased substantially (2001: 296540 cases, 2005: 11507 cases). The reason for this reduction was not mentioned in detail, however this is probably due to the effect of mass drug administration by the North Korean government supported by South Korea. We suspect that the population structure of *P. vivax* in North Korea was changed dramatically and that these predominant “old” haplotypes were eliminated completely or became very minor in both the North Korean and South Korean populations. We suspect that the South Korean *P. vivax* population is a subpopulation of the North.

Our previous genetic epidemiological analyses of the South Korean *P. vivax* population using antigenic molecules [21]–[25] and the mitochondrial genome [39] showed that there were 2 types (or groups) of parasite populations in the endemic area. In these previous studies we examined groups of isolates collected from vivax malaria patients in the DMZ in 1997 [21], 1998 [22]–[24], 1999 [39]. In the present study, we examined isolates collected from patients in the DMZ between 1994 and 2008 using 10 highly polymorphic microsatellite loci. Once again, we observed two types of parasite populations (Fig. 4). However, some other haplotypes (clones) have been observed in the endemic area since 1998. The new haplotypes were genetically different to the 2 major groups that have been transmitted since the beginning of the re-emergence (Fig. 4). This finding was consistent with the results of analyses by Choi et al. using the DNA sequences of 2 antigenic molecules (circumsporozoite protein, merozoite surface protein-1) of isolates collected in the DMZ from 1996 to 2007 [40]. They also reported that new genotypes have been observed since 2000 and that the new genotypes had been rapidly disseminated in the endemic area.

The genetic differences between the 2 major groups and the new haplotypes in our data suggested two possibilities: the new haplotypes could have arisen in the DMZ in South Korea through recombination between existing clones in the population; or their emergence could be attributed to a continual introduction of *P. vivax* from other population sources, probably from North Korea. The present study suggested a low recombination rate in the South Korean population and would seem to indicate that the latter possibility is more likely.

A less likely possibility is that all of the isolates examined in this study were continually introduced from North Korea because all the isolates were collected from South Korean soldiers who served in the DMZ. These patients were normally treated by chloroquine within 4 days of the onset of the symptoms, and then treated by primaquine as a radical cure. The recurrence rate (both new infection and relapse may be included) of vivax malaria among them is 1.6% (62 cases of 3881 cases) and the definitive relapse rate is only 0.2% (8 cases of 3881 cases) [41]. In addition, the incubation period of *P. vivax* on the Korean peninsula is very long (8 months to 13 months) [14] and the transmission is mainly in summer [38]. Moreover, the period of conscription is about 2 years. Therefore, it might have been very difficult to transmit continuously among the South Korean soldiers in the DMZ, leading to the high recombination rate of the genome within the parasite population of the study area.

There are a number of sampling limitations to the present study. The number of isolates per year was relatively small (2 to 14 isolates, average: 5.8 isolates/year), and the sample size during the years 2004–2008 was particularly small (2 to 7 isolates, average: 3.6 isolates/year). Moreover, all of the isolates used in this study were collected only from South Korean soldiers or veterans and not from civilians, whose proportion among vivax malaria patients in South Korea has been gradually increasing [18]. In order to overcome these limitations and more accurately estimate the current status of the parasite population in South Korea, it will be necessary to include new isolates collected from civilians in the endemic areas and to increase the sample size of recent years.

Although travel between South and North Korea is basically restricted and the malaria control programs in the two countries may not be the same, we suspect that the South Korean *P. vivax* population is a subpopulation of the North Korean population because the majority of malaria patients live near the border [42]. *Anopheles* mosquitoes can fly over the DMZ, and South Korean travelers are allowed to visit some parts of North Korea, such as Kaesong and Kumgang-san, which are very famous for sightseeing. Furthermore, from 2001 to 2009 the number of vivax malaria cases in North Korea ranged from twice as high as the number in South Korea to many times higher, indicating that the size of the parasite population in North Korea is probably larger. Thus, the inclusion of North Korean isolates in the analyses would greatly enhance the accuracy of the estimation of the parasite population structure and the transmission dynamics and provide a more complete picture of the *P. vivax* population in the Korean peninsula; unfortunately the feasibility of doing this is low.

In conclusion, molecular epidemiology using highly polymorphic DNA markers of the *P. vivax* population is a very useful tool for assessing the population structure and transmission dynamics of the parasites, the knowledge of which may lead to the effective control of vivax malaria in the respective endemic areas.
